# Proximity-dependent biotinylation detects associations between SARS coronavirus nonstructural protein 1 and stress granule–associated proteins

**DOI:** 10.1016/j.jbc.2021.101399

**Published:** 2021-11-11

**Authors:** Yevgeniy A. Gerassimovich, Samantha J. Miladinovski-Bangall, Kaitlin M. Bridges, Linkel Boateng, Lauren E. Ball, Homayoun Valafar, Anita Nag

**Affiliations:** 1 Natural Sciences and Engineering, University of South Carolina Upstate, Spartanburg, South Carolina, USA; 2 Department of Computer Science and Engineering, University of South Carolina, Columbia, South Carolina, USA; 3 Department of Cell and Molecular Pharmacology, Medical University of South Carolina, Charleston, South Carolina, USA

**Keywords:** SARS-CoV, SARS-CoV-2, nsp1, stress granule, BioID2, BSA, bovine serum albumin, α-CoV, α-coronavirus, β-CoV, β-coronavirus, eIF, eukaryotic translation initiation factor, FDR, false discovery rate, G3BP1, Ras GTPase-activating protein SH3 domain–binding protein 1, HA, hemagglutinin, HEK293, human embryonic kidney 293 cell line, MERS-CoV, Middle Eastern respiratory syndrome coronavirus, nsp1, nonstructural protein 1, SARS-CoV, severe acute respiratory syndrome coronavirus, SARS-CoV-2, severe acute respiratory syndrome coronavirus 2, SG, stress granule

## Abstract

The nonstructural protein 1 (nsp1) of severe acute respiratory syndrome coronavirus and severe acute respiratory syndrome coronavirus 2 is a critical viral protein that suppresses host gene expression by blocking the assembly of the ribosome on host mRNAs. To understand the mechanism of inhibition of host gene expression, we sought to identify cellular proteins that interact with nsp1. Using proximity-dependent biotinylation followed by proteomic analyses of biotinylated proteins, here we captured multiple dynamic interactions of nsp1 with host cell proteins. In addition to ribosomal proteins, we identified several pre-mRNA processing proteins that interact with nsp1, including splicing factors and transcription termination proteins, as well as exosome, and stress granule (SG)–associated proteins. We found that the interactions with transcription termination factors are primarily governed by the C-terminal region of nsp1 and are disrupted by the mutation of amino acids K164 and H165 that are essential for its host shutoff function. We further show that nsp1 interacts with Ras GTPase-activating protein SH3 domain–binding protein 1 (G3BP1) and colocalizes with G3BP1 in SGs under sodium arsenite–induced stress. Finally, we observe that the presence of nsp1 disrupts the maturation of SGs over a long period. Isolation of SG core at different times shows a gradual loss of G3BP1 in the presence of nsp1.

Severe acute respiratory syndrome coronavirus (SARS-CoV) and severe acute respiratory syndrome coronavirus 2 (SARS-CoV-2) are enveloped viruses containing a long, single, and positive-stranded RNA genome (1, 2, 3). Once the viral genome is released into host cells, the viral RNA, which is capped and polyadenylated, undergoes translation to produce two polyproteins containing 16 nonstructural proteins (nsps) that help in viral replication and propagation. Nsp1 is the N-terminal cleavage product of the polyprotein that lacks any known viral homologs.

The family Coronaviridae is classified into four genera: alpha coronavirus, beta coronavirus, gamma coronavirus, and delta coronavirus. SARS-CoV and SARS-CoV-2, along with the Middle Eastern respiratory syndrome coronavirus (MERS-CoV), are classified as β-coronaviruses (β-CoV), whereas human coronaviruses (HCoV-HKU1, HCoV-NL63, etc.) that cause mild cold-like symptoms are classified as α-coronaviruses (α-CoV) (4, 5). The α-CoVs and β-CoVs are primarily found in mammals. Interestingly, only mammalian coronaviruses (α-CoVs and β-CoVs) possess nsp1 responsible for stalling ribosome assembly and degrading host mRNAs, a process known as host shutoff. Even though nsp1 of the two SARS coronaviruses (SARS-CoV and SARS-CoV-2) show 84% sequence identity and 91% similarities, sequence homology with MERS-CoV nsp1 is only about 40%. In addition, no human protein shows a high degree of sequence homology to nsp1.

Nsp1 is a small (180 amino acid) protein containing a six-stranded β barrel-like structure whose one opening is covered by an α-helix (6, 7, 8). However, the rest of the protein is structurally flexible, which allows the protein to adopt different structures as it binds to host factors. For example, mapping of only the C-terminal region of nsp1 into the cryo-electron microscopy structure of the nsp1-bound 40S complex determined that the structurally undefined C-terminal domain of the protein folds into a defined helix–turn–helix structure to bind the mRNA binding site of the 40S ribosome (9, 10). In this experiment, Schubert *et al.* (9) studied thermodynamically stable complexes between bacterially expressed nsp1 and human embryonic kidney 293 (HEK293) cell lysate. The authors identified nsp1 not only in the 43S preinitiation complex but also in the 80S ribosomal complexes even though nsp1 disrupts the assembly of 80S ribosome on host mRNAs (9, 10). Identification of nsp1 bound to the 40S and 80S complexes by cryo-electron microscopy only captures the proteins stably bound to nsp1 but does not identify all the dynamic interactors of nsp1 during its host shutoff activity.

Mammalian yeast-two-hybrid experiments of individual SARS-CoV ORFs did not identify an interaction between nsp1 and another SARS-CoV protein (11, 12). Expression of nsp1 alone in HEK293 and HeLa cells consistently showed downregulation of host gene expression, which is also reproduced in the cell-free system (13, 14, 15, 16, 17). Therefore, the host shutoff function of nsp1 must be executed through its interaction with multiple host proteins. Genome-wide interactions of coronavirus proteins have been identified using MS in cells expressing SARS-CoV-2 proteins (18), in-cell stable isotope labeling using subgenomic SARS-CoV, and using biotin labeling of mouse hepatitis virus–infected cells (19, 20, 21). While these studies provided valuable information, none of them focused exclusively on nsp1 and compared the binding of nsp1 of SARS-CoV and SARS-CoV-2 to host proteins. In this investigation, we explored proximity-dependent biotinylation of neighboring molecules using a BioID2 fusion to nsp1, which allows biotinylation and subsequent purification of neighboring proteins (22, 23, 24, 25, 26, 27, 28, 29). This method enables labeling in live cells to capture transient events in the native environment and allows the identification of proteins that are part of insoluble structures and interactions that do not endure protein isolation under typical immunopurification techniques. Here, we focused on identifying pathways that interact with nsp1 to facilitate or inhibit host shutoff. We further report that several pre-mRNA processing proteins and stress granule (SG)–associated factors were identified in addition to proteins involved in mRNA translation.

## Results

### BioID2-dependent biotinylation of nsp1 interacting proteins and their identification

**Figure 1:**
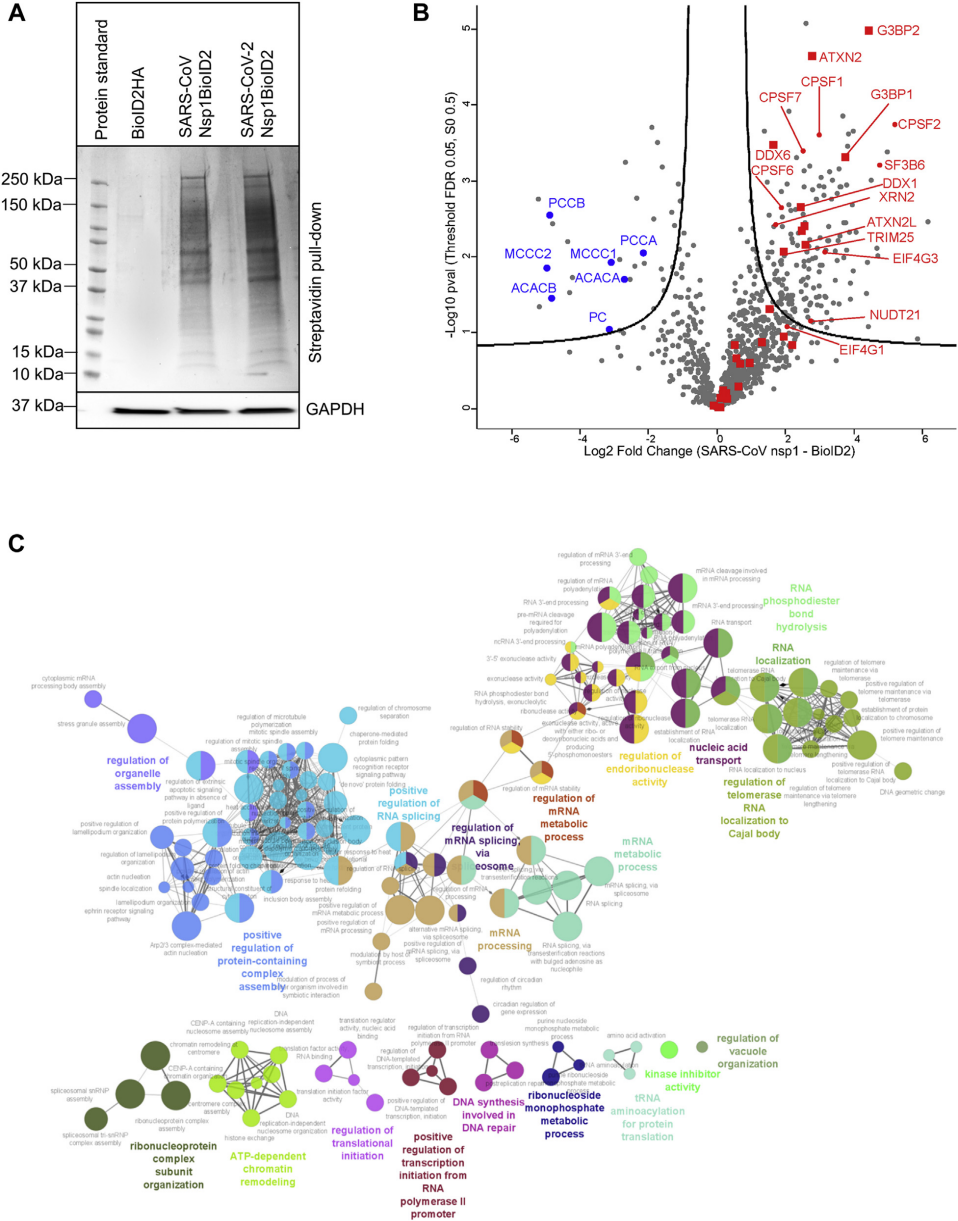
**Biotinylation of proximal proteins using nsp1-BioID2 and detection by LC–MS/MS reveals proteins involved in RNA processing and stability.***A*, cells expressing BioID2 fusion protein were treated with biotin, and biotinylated proteins were isolated with streptavidin beads, separated in an SDS gel, and stained with Coomassie staining (*top*). Equal loading was confirmed by Western blot analysis of GAPDH (*bottom*). *B*, volcano plot showing statistically enriched proteins present in nsp1 samples of both SARS-CoV and SARS-CoV-2 (*right*) relative to BioID2 (*left*). The threshold lines represent a permutation-based FDR <0.05 and S0 fold change parameter of 0.5. Endogenously biotinylated carboxylases enriched in the control are in *blue*. Proteins annotated with Gene Ontology (GO) terms associated with stress granules (stress granule assembly [GO: 0034063], cytoplasmic stress granule [GO: 1903608]) are indicated with *red-filled squares*. *C*, ClueGo analysis was conducted in Cytoscape using strong nsp1 interactors to visualize major pathways (GO_BiologicalProcess-EBI-UniProt-GOA-ACAP-ARAP_08.05.2020_00h00). FDR, false discovery rate; nsp1, nonstructural protein 1; SARS-CoV, severe acute respiratory syndrome coronavirus; SARS-CoV-2, severe acute respiratory syndrome coronavirus 2.

To capture proteins that dynamically interact with nsp1 in the native environment of the cell and the cellular pathways that facilitate the function of nsp1s, we performed proximity labeling using an engineered biotin ligase (BioID2) fused to nsp1 in HEK293 cells (Fig. S1). BioID2 is a biotin ligase from *Aquifex aeolicus* containing a mutation in its biotin ligase active site (R40G) that allows BioID2 to promiscuously biotinylate proximal proteins (23). We isolated biotinylated proteins from cells expressing the fusion protein using streptavidin-coated magnetic beads (Fig. 1*A*) and subjected these affinity-enriched proteins to proteomic analyses by LC–MS/MS. Duplicate experiments were performed to compare proteins biotinylated with nsp1-BioID2 from both SARS coronaviruses (SARS-CoV and SARS-CoV2) to proteins identified in the BioID2-hemagglutinin (HA) control. Proteins were filtered to retain those quantified in all biological replicates, yielding 946 proteins. To identify proteins in proximity to nsp1, log2-transformed intensities of proteins from nsp1-BioID2 and BioID2-HA were compared with a *t* test. The threshold (false discovery rate [FDR] <0.05, S0 fold change parameter of 0.5) was set using endogenously biotinylated carboxylases (*blue*), which were enriched in the negative control as expected (Fig. 1*B*, *volcano plot*). These and other proteins, such as histones, exhibiting a higher relative abundance in the BioID2-HA control are known to be enriched with streptavidin beads and the HA epitope (30).

Using this criterion, 113 proteins were enriched from nsp1-BioID2 reactions by at least fourfold, and 11 were enriched at least larger than eightfold as compared with the control (protein lists are provided in Tables S1–S3). A separate volcano plot showed differential interactions of proteins in SARS-CoV nsp1 and SARS-CoV-2 nsp1 samples (Fig. S2 and Table S4). Six proteins were identified only in the SARS-CoV nsp1 sample, whereas 17 proteins were only identified in the SARS-CoV-2 nsp1 sample. In this study, we focused on proteins that were found to interact with nsp1 of both SARS-CoV and SARS-CoV-2.

Major cellular pathways involving 113 strong interactors of nsp1 were visualized using the ClueGO plug-in in Cytoscape software (INSERM, AVENIR Team, Integrative Cancer Immunology, U872) (Fig. 1*C*) (31). Major pathways observed include pre-mRNA splicing and RNA metabolism–related complexes. This network also revealed proteins involved in SG assembly (Fig. 1, *B* and *C*). An extended analysis of MS data to capture putative proteins in proximity to nsp1 (see the Experimental procedures section) revealed multiple pathways related to transcription and RNA stability (Table S5). Consistent with the binding between nsp1 and the 40S ribosome, we identified several 40S ribosomal proteins (RPS7, RPS9, RPS16, RPS17, RPS23, and RPS26) along with eukaryotic translation initiation factors (eIF1D, eIF1G, eIF3I, and eIF3G). The recently published cryo-electron microscope structure also captured nsp1 with the 80S ribosome (9, 10). Consistent with this interaction, we identified multiple 60S ribosomal proteins (Table S5). While the presence of translation-related proteins confirmed our method, which is consistent with the established function of nsp1 (14, 15), we pursued validation of other pathways that may reveal any new role of nsp1 in disrupting host mRNA stability.

**Figure 2:**
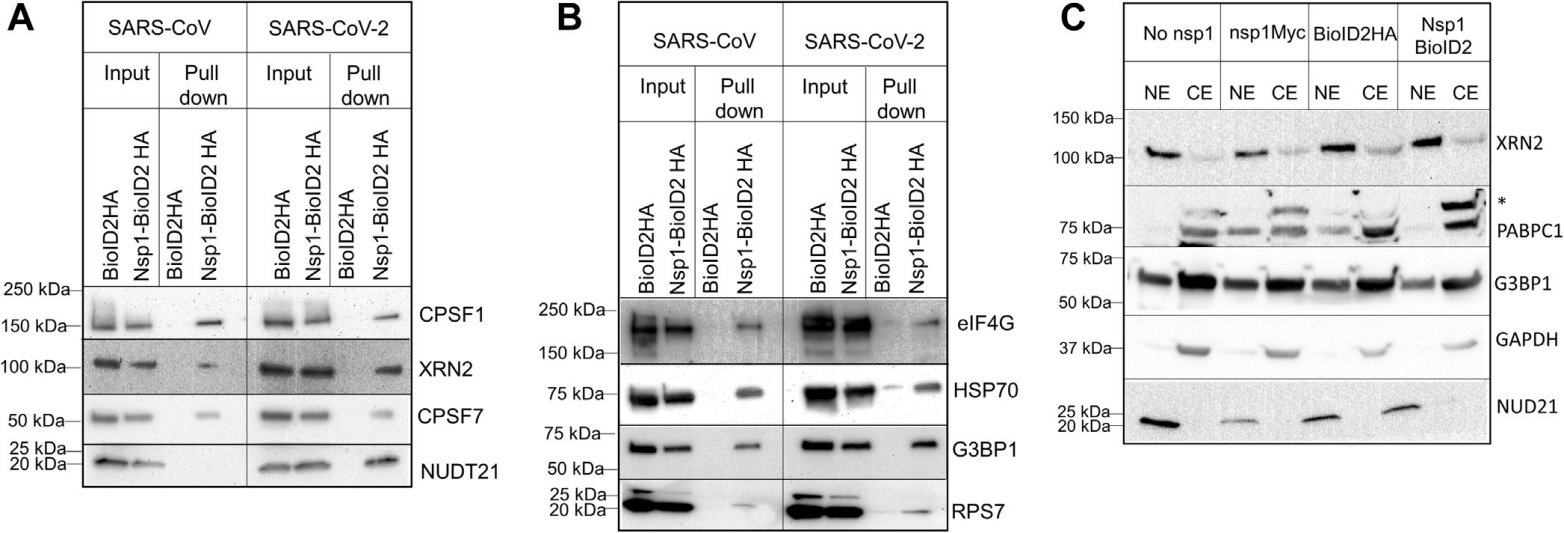
**Verification of MS data with Western blot analysis shows the association of nsp1 with both nuclear pre-mRNA processing proteins and stress granule proteins.***A*, streptavidin pull down was conducted from cells expressing BioID2-fused nsp1 in the presence of 100 μM biotin followed by immunoblot of nuclear proteins involved in pre-mRNA cleavage and polyadenylation. *B*, streptavidin pull down and immunoblot of proteins involved in heat shock, stress, and translation from BioID2-fused nsp1-expressing cells grown in the presence of 100 μM biotin. *C*, analysis of the localization of nuclear proteins in the presence of nsp1. Either nsp1-Myc (SARS-CoV) or nsp1-BioID2 (SARS-CoV-2) were expressed in HEK cells for 24 h followed by the separation of nuclear and cytoplasmic fractions. Each fraction was analyzed in an immunoblot using antibodies against nuclear pre-mRNA processing proteins (NUDT21 and XRN2), cytoplasmic proteins (G3BP1 and PABPC1), and a control (GAPDH). HEK, human embryonic kidney cell line; nsp1, nonstructural protein 1; SARS-CoV, severe acute respiratory syndrome coronavirus; SARS-CoV-2, severe acute respiratory syndrome coronavirus 2.

Interestingly, the largest cluster of proteins identified includes proteins involved in mRNA transcription, splicing, and 3′-end processing (Fig. 1*C*). Proteins identified by MS were verified using Western blot of translation initiation factor (eIF4G), heat shock protein 70, SG factors including the Ras GTPase-activating protein SH3 domain–binding protein 1 (G3BP1), and pre-mRNA processing proteins (CPSF1, CPSF7, NUDT21, and XRN2) shown in Figure 2, *A* and *B*. While these proteins were identified by MS in both SARS-CoV and SARS-CoV-2 nsp1 samples, some of the pre-mRNA processing proteins (NUDT21 and XRN2) showed enhanced affinity toward SARS-CoV-2 nsp1 in the immunoblot (Fig. 2*A*).

Since SARS-CoV and SARS-CoV-2 are cytoplasmic viruses and nsp1 is primarily located in the cytoplasm (although a small amount of nsp1 is detected in the nucleus *via* immunofluorescence), identification of nuclear pre-mRNA processing proteins (mRNA cleavage and splicing) was unexpected. These proteins include multiple subunits of the cleavage-polyadenylation specificity factors (CPSF1, CPSF2, CPSF4, CPSF5, and CPSF7) and the 5′ to 3′ exonuclease (XRN2) involved in nuclear pre-mRNA cleavage and transcription termination (Fig. 2 and Table S1) (32, 33, 34). Moreover, several proteins (UPF3B and eIF4A3) involved in nonsense-mediated decay were identified by MS. Confirming our previous observation that nsp1 interacts with the nuclear pore complex (35), we found proteins involved in mRNA transport (Nup50 and RAE1) in our MS data (Table S5). In addition, in accordance with the current publications (19, 36), we identified nucleolin, a protein involved in mRNA stability, in the MS results (data not shown). Since we demonstrated disruption in nuclear–cytoplasmic transport in our earlier work (35), which was recently confirmed by Zhang *et al.* (36), we inquired if the interaction of nsp1 with nuclear pre-mRNA processing proteins is due to their altered localization to the cytoplasm. Fractionation of nuclear and cytoplasmic proteins in the presence and absence of nsp1 was performed, and nuclear pre-mRNA processing proteins were analyzed by Western blot. As shown in Figure 2*C*, we did not find any significant alteration in the localization of CPSF1, CPSF7, NUDT21, and XRN2 in the cytoplasm in the presence of nsp1. GAPDH served as a control as it is exclusively present in the cytoplasmic fraction. It is worth pointing out that nsp1 of another β-coronavirus, MERS-CoV, has been shown to mark mRNAs in the nucleus for their degradation in the cytoplasm. This observation was recorded using electroporated mRNAs that are not subjected to a similar host shutoff effect as endogenous mRNAs (37). Similarly, the influenza virus host shutoff protein PA-X associates with nuclear pre-mRNA processing proteins (38).

### Transient interaction of pre-mRNA processing and SG proteins are altered by C-terminal mutation of nsp1

**Figure 3:**
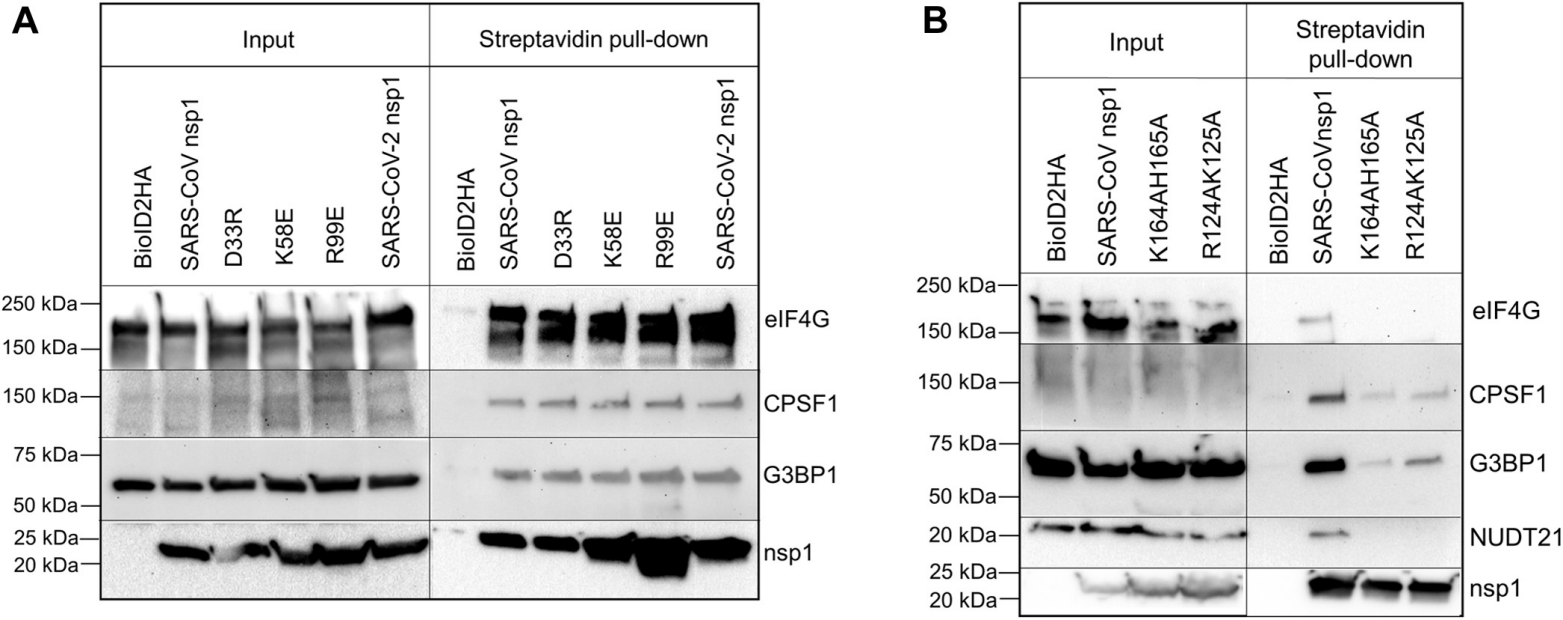
**Proximity-dependent labeling and pull down identify the association between mutant nsp1 and cellular proteins.***A*, BioID2-fused nsp1 proteins carrying mutations in the middle region of nsp1 were expressed, treated with 100 μM biotin for 16 h, and biotinylated proteins were isolated with streptavidin-coated magnetic bead. These proteins were separated in SDS gel followed by the analysis of specific protein association using immunoblot. *B*, the aforementioned experiment was repeated with nsp1 carrying mutations in the structurally flexible C terminus of the protein followed by immunoblot. C-terminal mutations weaken association by CPSF1, NUDT21, and eIF4G. nsp1, nonstructural protein 1.

Nsp1 is an inherently flexible protein with a β-barrel and an α-helix structure in the middle of the protein, whereas both the C and N terminus are structurally flexible (6, 7). The inherently flexible C-terminus region of the protein folds to accommodate a helix–turn–helix structure to bind the mRNA binding pocket of the 40S ribosome rendering it incapable of binding the 60S ribosome and initiating translation (9). A mutation in the flexible C terminus of nsp1 (K164A and H165A) disrupts the ability of nsp1s to bind in the mRNA binding pocket of the 40S ribosome and inhibits its host shutoff activity. Mutations in the nearby R124 and K125 to alanines do not affect its ability to dampen translation but eliminate the ability of nsp1s to trigger RNA cleavage. In addition to the K164A and H165A mutations, Jauregui *et al.* (39) conducted a thorough investigation of amino acid side chains on the surface of nsp1 that attenuate (K58E and R99E) or accentuate (D33A) its host shutoff activity, potentially through its interaction with host factors. Using BioID2-mediated biotinylation followed by Western blot, we examined the proximity-dependent interaction of several pre-mRNA processing proteins and SG-associated factors with nsp1 mutants (Fig. 3, *A* and *B*). As expected, the mutation in the C terminus of nsp1—known to disrupt its interaction with the translational machinery—prohibited the interaction of nsp1s to the cap-binding protein eIF4G. As a control, we investigated reported mutations in the middle region of the protein that are known to enhance (D33R) or attenuate (K58E and R99E) the ability of nsp1s to suppress protein translation (39). Mutations at D33, K58, and R99 residues did not alter the interaction of nsp1s with pre-mRNA cleavage and processing factors. However, mutations R124A, K125A, and K164A, H165A disrupted its interaction with NUDT21. On the contrary, SG-associated protein, G3BP1, and cleavage-polyadenylation protein, CPSF1, retained modest interaction with nsp1 upon mutations of the key C-terminal amino acids (Fig. 3*B*). These results indicate that the C-terminal region of nsp1 is important for its interaction with not only the ribosome but also to pre-mRNA processing and SG-associated proteins. It is important to note that the aforementioned experiments specifically capture dynamic interactions between nsp1 and cellular proteins, and proteins identified by this method may not always represent thermodynamically stable interactions.

### SGs incorporate both nsp1 and G3BP1

SGs are cytoplasmic foci that develop when translationally stalled mRNAs form membrane-less granules, triggered by different types of cellular stress (oxidative stress, amino acid deprivation, viral RNA, etc.) (40, 41). This granule formation is aided by multiple SG proteins that aggregate using their inherently disordered regions. It has been demonstrated that the interaction between SG protein, G3BP1, and cap-binding component, eIF4G, is essential for SG assembly (42).

**Figure 4:**
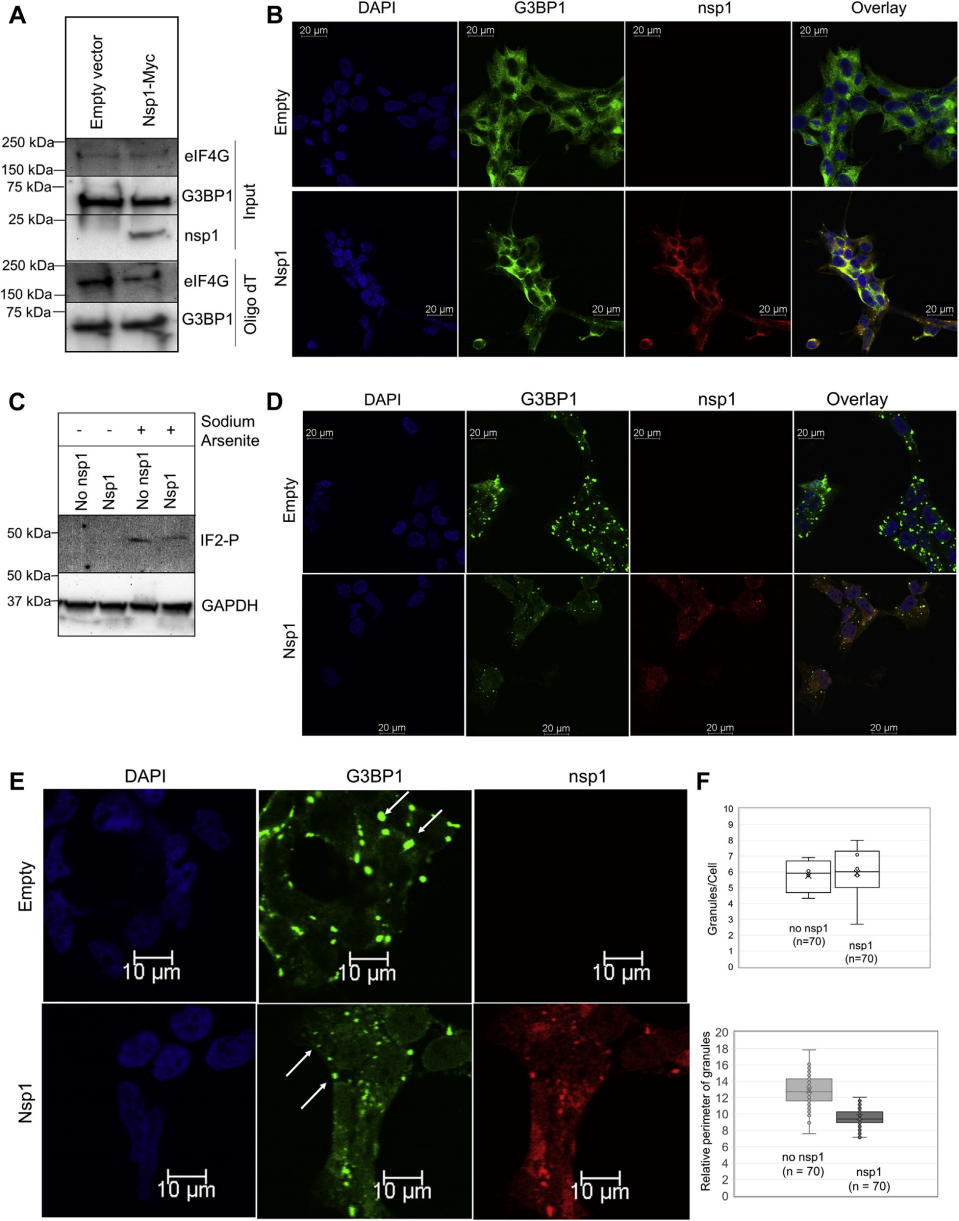
**Sodium arsenite–induced stress granule formation in the presence and absence of nsp1 identifies colocalization of G3BP1 and nsp1.***A*, association of eIF4G and G3BP1 to mRNA was analyzed in the presence of nsp1 from cells expressing nsp1-Myc. OligodT pull down was conducted from cells with and without nsp1, followed by immunoblot analysis of G3BP1 and eIF4G pulled down by OligodT. *B*, HEK cells were transfected with pCAGGS-nsp1-Myc. Cells were fixed after 24 h, and protein localization was observed using anti-G3BP1 and anti-nsp1 antibodies. Images were captured using Leica TCS SP8 confocal microscope with a 40× objective. *C*, cells undergoing sodium arsenite treatment were analyzed for phosphorylation of eIF2α and GAPDH (control) using immunoblot. *D*, stress was induced by 0.5 mM NaAsO_2_ for 40 min, and cells were fixed and probed with anti-G3BP1 and anti-Myc antibodies. *E*, a zoomed-in representation of stress granules in cells with and without nsp1-Myc. *F*, the quantification of stress granules was done using ImageJ software. The relative perimeter was calculated in micromolar (3.52 pixel/μm). eIF, eukaryotic translation initiation factor; G3BP1, Ras GTPase-activating protein SH3 domain–binding protein 1; HEK, human embryonic kidney cell line; nsp1, nonstructural protein 1.

In order to investigate if nsp1 disrupts the interaction of G3BP1 and eIF4G with mRNA, cells were grown in the presence and absence of nsp1, and the cellular extract was collected. OligodT beads were used to isolate mRNA and proteins associated with it. Western blot analysis of mRNA-associated proteins did not show any significant difference in G3BP1 and eIF4G levels, confirming that nsp1 by itself does not disrupt the interaction of G3BP1 and eIF4G to mRNAs (Fig. 4*A*). Since nsp1 was found to be associated with multiple SG components (G3BP1, eIF4G, and ATXN2L) in our MS result, we inquired if nsp1 localizes with SG-associated proteins upon induction of external stress. HEK293 cells were grown in the presence and absence of nsp1 expression and were subsequently stained with an anti-G3BP1 antibody. In unstressed cells, G3BP1 remains uniformly distributed in the cytoplasm in the presence of nsp1 (Fig. 4*B*). Treatment of these cells with 0.5 mM sodium arsenite (NaAsO_2_) triggers oxidative stress and induces phosphorylation of eIF2α in both samples (Fig. 4*C*). Upon induction of stress, G3BP1 localization alters from a uniform distribution in the cytoplasm to bright punctuated dots representing SGs (Fig. 4*D*). In the presence of nsp1, we observed both nsp1 and G3BP1 colocalize in these SGs. Calculation of the perimeter of SGs with ImageJ (43) showed, on average, that smaller punctuated structures accumulate in nsp1-containing cells compared with cells lacking nsp1 (Fig. 4, *E* and *F*).

**Figure 5:**
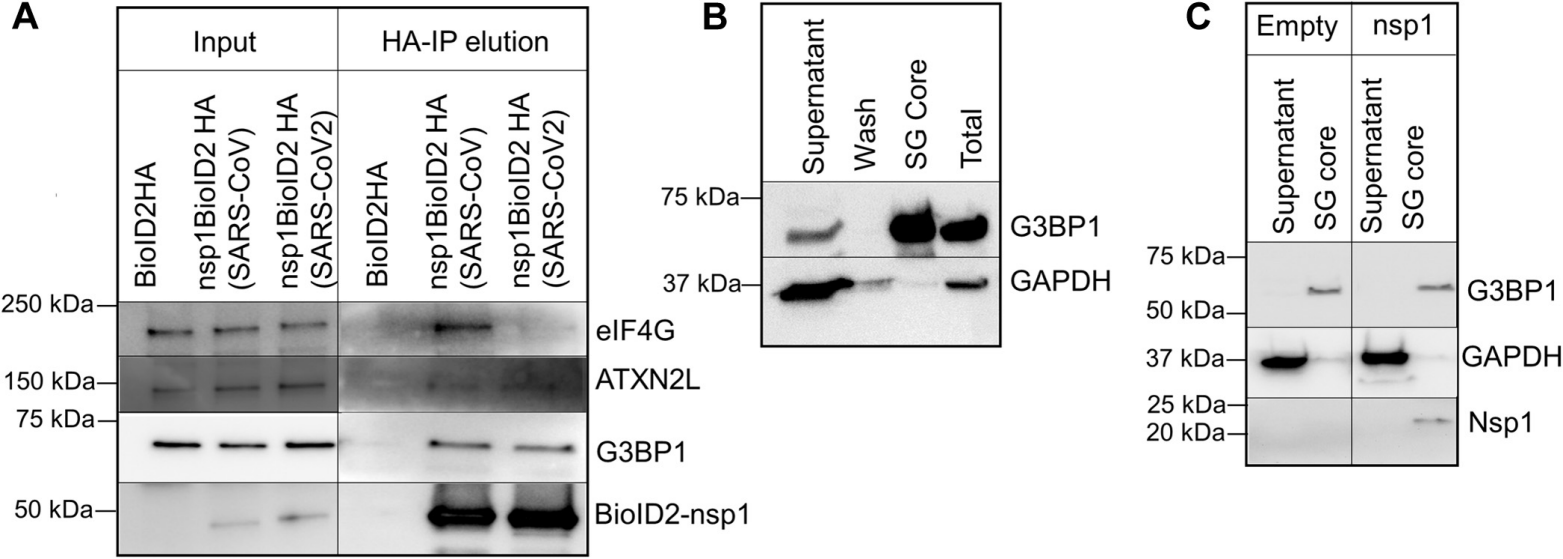
**Interaction between nsp1 and stress granule–associated proteins is identified using immunoprecipitation of nsp1.***A*, nsp1-BioID2HA was pulled down from cells expressing nsp1-BioID2HA using anti-HA magnetic beads. The protein complex was eluted with an HA peptide and was analyzed in an immunoblot using anti-G3BP1 and anti-eIF4G antibodies. *B*, the stress granule core was isolated using the published literature, and the presence of G3BP1 in the core and GAPDH in the remaining cytoplasmic fraction (supernatant) was verified using immunoblot. *C*, the stress granule isolation step was repeated using cells expressing SARS-CoV2 nsp1-V5. Subsequent immunoblots show nsp1 and G3BP1 in the stress granule core, whereas GAPDH is present in the remaining cytoplasmic fraction (supernatant). eIF, eukaryotic translation initiation factor; G3BP1, Ras GTPase-activating protein SH3 domain–binding protein 1; HA, hemagglutinin; nsp1, nonstructural protein 1; SARS-CoV2, severe acute respiratory syndrome coronavirus 2.

It has been recently reported that upon viral infection, especially by RNA viruses, SGs not only protect translationally stalled mRNAs but also accumulate several type I interferon–stimulated gene products as an antiviral response (44). In a recent report, Zheng *et al.* (45) also observed that knockdown of G3BP1 significantly diminishes viral replication. Since BioID2-based assay only captures dynamic interactions because of the proximity of proteins, we inquired if nsp1 binds to G3BP1 in cells and also isolates with the core of SGs upon stress induction. To address the first question, we conducted an anti-HA pull down of nsp1 from cytoplasmic extract followed by HA peptide elution of the protein complex. We observed the presence of several SG-associated proteins including G3BP1 in the nsp1 pull down (Fig. 5*A*). EIF4G also showed an interaction with SARS-CoV nsp1, which is significantly weakened for SARS-CoV2 nsp1. To address the latter question, we isolated SGs from cells after inducing 30 min of stress following an established protocol (46, 47). Protein analysis of isolated SG core verified the presence of G3BP1, whereas GAPDH was excluded from this complex, as expected (Fig. 5*B*). When the experiment was repeated using nsp1-expressing cells, nsp1 was detected in the SG core along with G3BP1 (Fig. 5*C*). We concluded that nsp1 interacts with G3BP1 and accumulates in the SG.

### Nsp1 modifies SG composition

Several viruses, including Semliki Forest virus and poliovirus, perturb SG formation (48, 49). Since the poliovirus induces proteolytic cleavage of G3BP1, we investigated proteolytic cleavage of G3BP1, eIF4G, and the phosphorylation level of eIF2α after 120 min of NaAsO_2_ treatment in cells expressing nsp1. We did not find any significant change in G3BP1 and eIF4G, whereas eIF2α showed a modest decrease in the phosphorylation level (Fig. S3).

**Figure 6:**
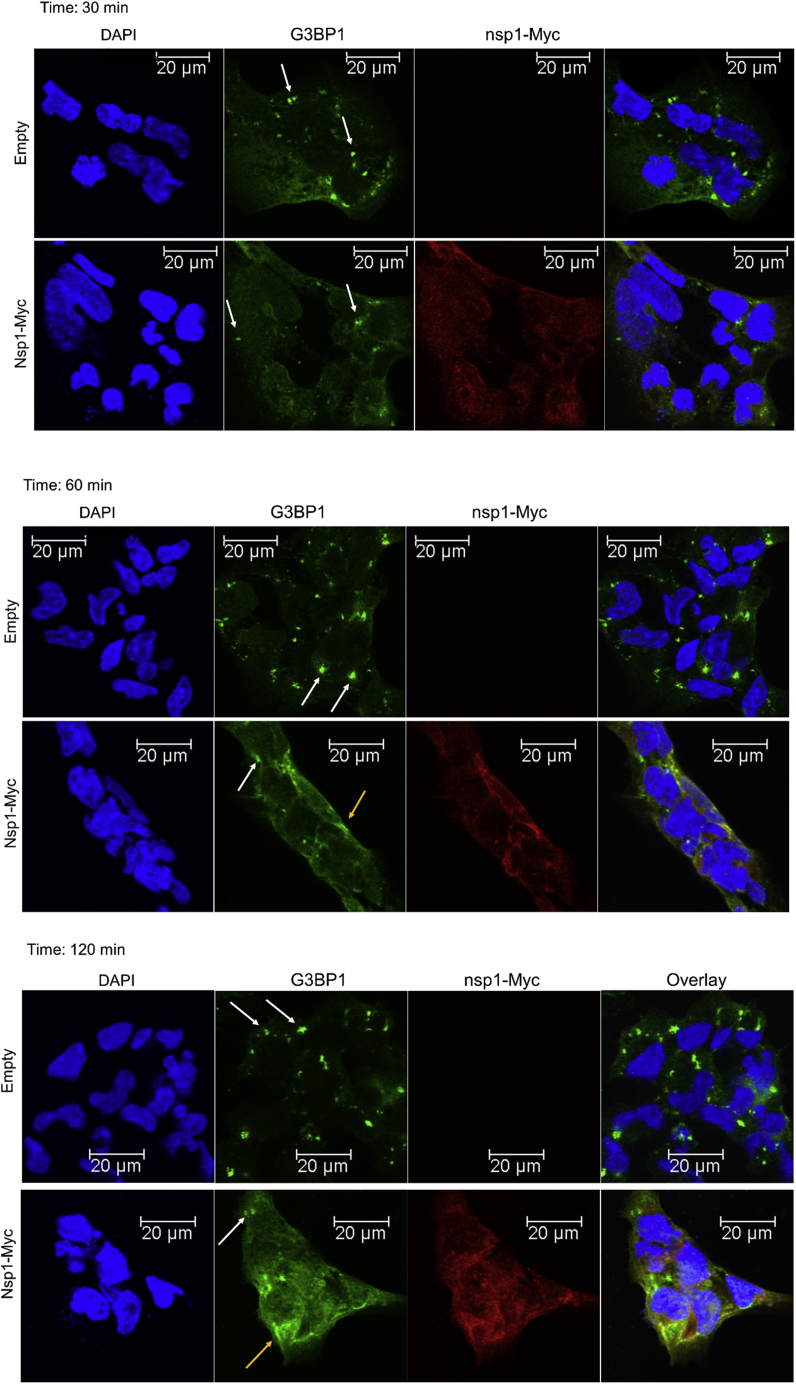
**Immunofluorescence of G3BP1 to observe stress granule assembly in the presence and absence of nsp1 over 2 h of oxidative stress.** HEK cells were transfected with pCAGGS-nsp1-Myc expression plasmid. Twenty-four hours later, sodium arsenite–mediated stress was induced for (*top*) 30, (*middle*) 60, and (*bottom*) 120 min. Cells were fixed and stained with anti-G3BP1 and anti-Myc antibodies. The image was collected using Leica TCS SP8 confocal microscope using 40× objective. The *white arrow* shows stress granules, and the *yellow arrow* shows diffused localization of G3BP1. G3BP1, Ras GTPase-activating protein SH3 domain–binding protein 1; HEK, human embryonic kidney; nsp1, nonstructural protein 1.

**Figure 7:**
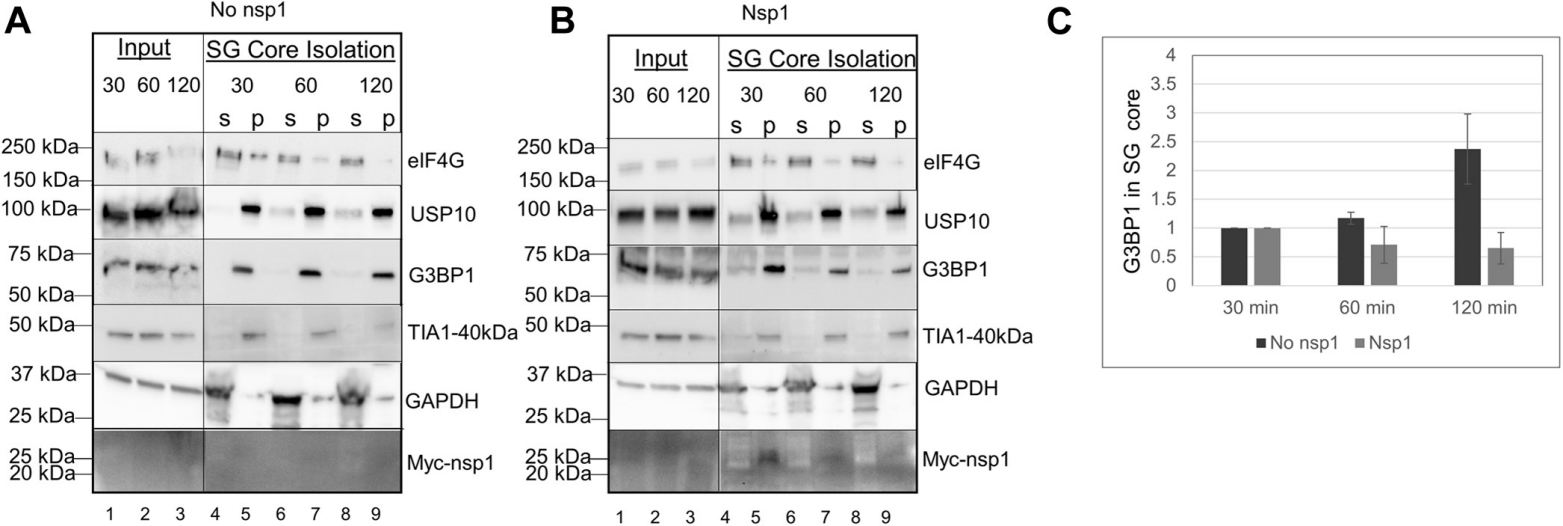
**Isolation of stress granules at 30, 60, and 120 min after NaAsO**_**2**_**-induced stress shows a decrease in G3BP1 after 2 h of oxidative stress.** Stress granules were isolated from cells without (*A*) and with SARS-CoV-nsp1-Myc (*B*) followed by an immunoblot of stress granule–associated proteins (eIF4G, USP10, G3BP1, TIA1-40kDa), nsp1 (Myc), and the control protein (GAPDH). Ten percent of the total and supernatant were loaded. Because of the low level of nsp1 expression in the cell, it is not visible in the 10% total input. *C*, ImageJ was used to quantify G3BP1 association to stress granules at 30, 60, and 120 min from three experiments. G3BP1 quantity was normalized to 1 for the 30 min of stress. G3BP1, Ras GTPase-activating protein SH3 domain–binding protein 1; nsp1, nonstructural protein 1; SARS-CoV, severe acute respiratory syndrome coronavirus.

It has been previously demonstrated that SG formation happens within the first 15 min of NaAsO_2_ treatment. The smaller size of SGs in nsp1-expressing cells may suggest a block in the aggregation of SGs to form a large assembly. Alternatively, a slower kinetic of SG formation may happen in the presence of nsp1. To distinguish between these two possibilities, we performed a time-course experiment using three different times of NaAsO_2_ treatment (30, 60, and 120 min). We observed that cells lacking nsp1 maintained SGs during 2 h of NaAsO_2_ treatment. Even though some SGs are still identified, a significant number of cells expressing nsp1 now contain dispersed and diffused localization of both G3BP1 and nsp1 after 2 h of treatment (Fig. 6, *middle and bottom panels*). Next, we isolated SG cores using published protocols (46, 47, 50) from cells that had undergone 30, 60, and 120 min of arsenite-induced stress and compared their protein compositions. The SGs isolated from cells without and with nsp1 show all known components of SG core (G3BP1, eIF4G, and TIA1) after 30 min of stress (Fig. 7, *A* and *B*). Quantification of G3BP1 showed a gradual increase in the accumulated level of the protein in SG core over 2 h (Fig. 7, *A* and *C*). In contrast, cells expressing nsp1 show a decrease in the level of both nsp1 and G3BP1 (Fig. 7, *B* and *C*). The decrease in both proteins could be due to the delocalization of both proteins as observed by the diffused immunofluorescence signal (Fig. 6, *yellow arrows*). The decrease in signal could be explained by the selective degradation of one or more proteins in the complex. We cannot rule out, at this point, that because of the host shutoff effect, nsp1-expressing cells lack a protein responsible for the integrity of SGs. We conclude that nsp1 interacts with G3BP1 in the SG and interferes with mature SG assembly.

## Discussion

The results presented here provide insights into the role of nsp1s in various cellular pathways, specifically in SG assembly. Since nsp1 can distinguish the viral RNA from host mRNAs, SG assembly may play a critical role in sorting host mRNAs and triggering them for degradation in processing bodies consistent with the existing knowledge of SG and P-bodies in RNA turnover (51, 52).

The proximity-dependent biotinylation assay, reported here, has captured the association between nsp1 and proteins involved in mRNA processing and stability. The association of nsp1 with the pre-mRNA processing complex is particularly interesting as any role of nsp1 in transcription regulation and maturation of mRNA has not been observed previously (Fig. 1). MERS-CoV nsp1 is known to modulate mRNAs in the nucleus so that only transcribed mRNAs undergo cleavage and degradation (37). Similarly, the influenza A virus endonuclease PA-X, also a host shutoff protein, interacts with multiple subunits of the CPSF complex (38). While other nsps of SARS-CoV-2 have been identified to modulate pre-mRNA splicing (52), our experiment uncovers an association between nsp1 and multiple members of pre-mRNA processing pathways using proximity labeling. Mainly, nsp1 associates with CPSF1, CPSF2, NUDT21, and XRN2, proteins that engage with the RNA polymerase II to induce the termination of pre-mRNA transcription and facilitate pre-mRNA cleavage (Figs. 2 and 4). It is important to note that the proximity-dependent labeling does not distinguish between a stable interaction and a more dynamic transient association.

The interaction between nsp1 and G3BP1 is consistent with the role of both proteins in mRNA stability and translation. To control host gene expression and stabilize viral RNAs for successful translation, viruses often modulate SG assembly to compartmentalize RNAs (48, 51, 53). Proximity-dependent labeling assay detected multiple members of the SG-associated proteins (Figs. 2 and 3). Furthermore, SG marker G3BP1 coimmunoprecipitates with nsp1 from cellular extract (Fig. 5). Coprecipitated G3BP1 represents a small portion of (∼5% or less) of the protein present in the cell leading us to believe that only a small population of G3BP1 interacts with nsp1 in the absence of stress. Upon induction of stress, nsp1 accumulates with known SG proteins G3BP1, eIF4G, and TIA1 in the isolated SGs (Fig. 7). The dynamics of mRNA accumulation in the SGs and processing bodies determine the stability of mRNAs in cells. Several viruses inhibit SG formation. NS1 protein of the influenza A virus blocks eIF2α phosphorylation and SG formation (54). In the case of nsp1, we did not notice a complete inhibition in eIF2α phosphorylation, but a modest change, suggesting that a step downstream of eIF2α may get perturbed after prolonged exposure to NaAsO_2_.

During the early stage of poliovirus infection, G3BP, eIF4G, and PABP associate with SGs (55, 56). At a later stage of viral infection, proteolytic cleavage of G3BP1, eIF4G, and PABP possibly releases these components from the SGs, resulting in dispersion of the SGs. It will be interesting to observe the SG assembly and disassembly in the context of coronavirus infection where other viral proteins and viral RNA may aid nsp1 (45). In cells expressing nsp1, the structural appearance of SGs changes after prolonged exposure to NaAsO_2_ (Figs. 4 and 6). This could be a result of post-translational modification of SG-associated proteins or a difference in the composition of SGs that prevents the maturation of SGs in the presence of nsp1. It will be interesting to evaluate these SG structures and their mRNA composition to identify specific RNAs subjected to SG accumulation in the presence of nsp1.

Several viruses target SG core protein G3BP1 to disrupt SG assembly (57). Herpes simplex virus protein ICP8 binds to G3BP and blocks SG formation. In Semliki Forest virus, nsP3 protein disrupts SG accumulation by blocking oligomerization of G3BP1. Both ICP8 and nsP3 proteins interact with G3BP1 using two FGDF sequences that disrupt SG assembly (57, 58). In contrast, nsp1 does not have a tandem FGDF sequence but carries a single FGDS sequence instead. This modified sequence will likely generate a weaker affinity toward G3BP1, as previously shown by the mutation of Phe to Ala (58). We performed a molecular dynamics simulation (59, 60, 61) of a single FGDF unit by replacing the Phe with Ser. Our calculation suggests a clear decrease in binding energy (−142 ± 13 kcal/mol to −78 ± 22 kcal/mol) upon substitution of Phe with Ser (Fig. S4). This weakened interaction may stabilize in the presence of other viral proteins during SARS-CoV infection. Recent advances in SARS coronavirus research revealed that nucleocapsid protein (N) and endoribonuclease (nsp15) also disrupt SG assembly (44, 45, 62, 63). Specifically, N protein interacts with G3BP1 to disrupt SG assembly during viral infection. Since SGs sequester multiple interferon-stimulated genes, it is conceivable that SARS coronaviruses use a multipronged strategy to circumvent antiviral defense of the cell employing both structural proteins and nsps. It will be important to know if nsp1 and N protein cooperate to enhance the effect or serve in parallel to impede antiviral defense.

Like other viruses that disrupt SG formation at different stages of viral replication, disruption of SG assembly in the presence of nsp1 will lead to weaker protection of stalled ribosomal complexes and degradation of mRNAs. It will be important to identify if nsp1 targets host mRNAs but spares viral RNAs by disrupting SG formation.

## Experimental procedures

### Plasmids and constructs

SARS-CoV nsp1 sequence used in this study was a kind gift (pCAGGS-nsp1-Myc) from Dr Shinji Makino (University of Texas Medical Branch). The SARS-CoV-2 nsp1 sequence was a kind gift from Dr Nevan Krogan (University of California San Francisco). BioID2-HA was purchased from Addgene (catalog no.: 74224). The pcDNA3-SARSCoV5-nsp1 plasmid was a gift from Dr Adi Dubash (Furman University, South Carolina) and was created in pcDNA V5 DEST (Invitrogen) using Gateway cloning. The nsp1 sequence was inserted in the EcoRI and BamHI sites. Mutations were created using primers shown in Table S6.

### Antibodies

Antibodies were used at a 1:500 to 1:1000 dilution for Western blot analyses. Antibodies were purchased from Santa Cruz Biotechnology: CPSF1 (G10, sc-166281), CPSF7 (A9, sc-393880), Xrn2 (H3, sc-365258), Hsp70 (B6, sc-7298), G3BP1 (H10, sc-363338), Rps7 (E1, sc-377317), NUDT21 (F5, sc-515766), eIF4G (A10, sc-133155), GAPDH (FL-335, sc-25778), and Sigma–Aldrich: phosphorylated eIF2α (SAB5700436), PABPC1 (HPA045423); Abcam ATXN2L (ab-99304), TIA1-40kDa (ab-263945); and Thermo Fisher Scientific: USP10 (PA5-52334) and V5-Tag (37-7500). The vertical and horizontal lines in the Western blot indicate where blots were spliced. Antibodies used in the immunofluorescence were obtained from Santa Cruz Biotechnology: Myc (9E10) AlexaFluor647 (sc-40AF647) and secondary antimouse AlexaFluor488 (sc-516176). Primary antibody was used at a 1:200 dilution for immunofluorescence.

### BioID2-mediated biotinylation and protein isolation

Methods were adapted from the Roux *et al.* (23) extraction of biotinylated proteins. In brief, HEK cells were grown under 5% CO_2_ at 37 °C to 50% confluency and in 10 cm plates. Cells in each plate were transfected with 4.8 μg of respective DNA to express BioID2-fused nsp1 protein using Jet Optimus transfection reagent (Genesee Scientific). Twenty-four hours post-transfection, cells were incubated with 100 μM biotin for 16 h. For the pull down, about 4 × 10^7^ cells were washed with ice-cold PBS. The cells were collected and lysed with 2.4 ml of BirA buffer I (50 mM Tris, pH 7.4, 500 mM NaCl, 0.4% SDS, 5 mM EDTA, and 2% Triton-X). After 5 min of incubation on ice, the cell lysate was sonicated two times, each for 30 s, at a 30% output level. An equal volume of BirA buffer II (50 mM Tris, pH 7.4) was added. The supernatant was collected after centrifugation of the lysate at 15,000*g* for 10 min. Each sample of supernatant was incubated with 400 μl Streptavidin beads overnight at 4 °C to collect biotinylated proteins. The streptavidin magnetic beads were washed four times with modified radioimmunoprecipitation assay buffer (50 mM Tris, pH 7.4, 150 mM NaCl, 1% NP-40, 0.5% sodium deoxycholate, and 0.1% SDS), one time with BirA buffer III (0.2% w/v SDS), once with BirA buffer IV (250 mM LiCl, 0.5% NP-40, 0.5% deoxycholate, 1 mM EDTA, and 10 mM Tris, and pH 8), and twice with BirA final buffer (50 mM Tris, pH 7.4). The proteins were eluted from the bead using SDS sample buffer and run on 4 to 20% precast gels (Bio-Rad). The gel was stained using a Colloidal Blue Staining Kit (Invitrogen). Lanes were fractionated into three sections for in-gel digestion and MS.

### MS

Proteins were reduced in 10 mM dithiothreitol (Thermo Fisher Scientific) at 55 °C and alkylated in 25 mM iodoacetamide (Thermo Fisher Scientific) for 30 min at room temperature in the dark. The protein was digested with trypsin (Sigma; 100 ng) overnight at 37 °C. Digestion was quenched by adding trifluoroacetic acid to a final concentration of 1%, and peptides were extracted from the gel and dried. Peptides were separated and analyzed on an EASY nLC 1200 System (Thermo Fisher Scientific) in line with the Orbitrap Fusion Lumos Tribrid Mass Spectrometer (Thermo Fisher Scientific) with instrument control software, version 4.2.28.14. Two micrograms of tryptic peptides were pressure loaded onto a C18 reversed-phase column (Acclaim PepMap RSLC, 75 μm × 50 cm [2 μm, 100 Å]; Thermo Fisher Scientific; catalog no.: 164536) and separated using a gradient of 5 to 40% B in 180 min (solvent A: 5% acetonitrile/0.2% formic acid; solvent B: 80% acetonitrile/0.2% formic acid) at a flow rate of 300 nl/min. Mass spectra were acquired in data-dependent mode with a high resolution (60,000) FTMS survey scan, mass range of *m/z* 375 to 1575, followed by tandem mass spectra (MS/MS) of the most intense precursors with a cycle time of 3 s. The automatic gain control target value was 4.0e^5^ for the survey MS scan. High-energy collision dissociation fragmentation was performed with a precursor isolation window of 1.6 *m/z*, a maximum injection time of 50 ms, and high-energy collision dissociation collision energy of 35%. Monoisotopic-precursor selection was set to “peptide.” Precursors within a mass tolerance of 10 ppm were dynamically excluded from resequencing for 15 s. Advanced peak determination was not enabled. Precursor ions with charge states that were undetermined, 1, or >5 were excluded.

Data acquired during 18 LC–MS/MS analyses were searched using MaxQuant, version 1.6.10.43, against a Human UniProt protein database (42,278 sequences; updated August 2020) including common contaminants. Fixed modification of cysteine with carboxyamidomethylation and variable oxidation of methionine and N-terminal acetylation were included. Two missed cleavages were permitted. Data were also searched against a reversed database, and a 1% FDR threshold was used for peptide spectral matches and protein identification. The precursor and fragment mass tolerances were 4.5 and 20 ppm, respectively. At least two peptides were required for protein quantification with at least one unique peptide. Intensity measurements were quantified and normalized by the MaxQuant label-free quantitation algorithm with matching between runs with a 0.7 min window to capture missing peptide peak intensity measurements (64). Data were processed in Perseus 1.6.0.8 (Max Planck Institute) using an affinity enrichment approach (65). Label-free quantitation normalized protein intensities were log2 transformed, and potential contaminants, reversed database hits, and proteins identified with less than two unique peptides were removed. Biotinylated proteins were filtered to retain those quantified in all biological replicates, and a two-sample and two-sided Student's *t* test was performed to compare nsp1-BioID to BioID-HA. The *p* value was adjusted for multiple hypothesis testing using a permutation-based FDR <0.05 with 250 randomizations (66). Gene Ontology terms, additional gene, and protein names were downloaded from the human UniProt database in October 2020. For downstream bioinformatics analysis, the dataset was expanded to include proteins with missing values in the BioID2-HA controls. Proteins were filtered to retain those quantified in all nsp1 replicates, and values missing in the BioID2-HA negative controls were imputed using a normal distribution with a downshift of 1.8 and width of 0.3. A *t* test was performed as described previously, and the *p* values, *q* values, and log2 fold changes are reported. To compare between SARS-CoV nsp1 and SARS-CoV-2 nsp1, proteins were filtered to retain those quantified in both replicates of either nsp1, and missing values were imputed as aforementioned. The *t* test *p* values and log2 fold changes are reported.

### OligodT pull down of RNA–protein complexes

OligodT pull down of RNA–protein complexes was performed from HEK293 cells grown on 10 cm plates that were transfected with pCAGGS-nsp1-Myc plasmid. Twenty-four hours post-transfection, RNA and proteins were crosslinked with 0.1% formaldehyde for 10 min at 37 °C and quenched with 0.25 M glycine at room temperature for 5 min. Cells were washed twice with PBS and collected in the lysis buffer (100 mM Tris, pH 7.5, 500 mM LiCl, 10 mM EDTA, 5 mM DTT, 0.1% NP-40, and 1× protease inhibitor). Cells were lysed with a 21-G needle five times and sonicated for 30 s at 30% output. The lysate was cleared by centrifugation, and the supernatant was incubated overnight at 4 °C with 200 μl oligo-dT magnetic bead precoated with bovine serum albumin. Beads were washed once with the lysis buffer and four times with wash buffer (10 mM Tris, pH 7.5, 150 mM LiCl, and 1 mM EDTA). Proteins were eluted using 1× SDS gel loading buffer and processed for SDS-PAGE followed by Western blot analysis.

### Immunofluorescence

Coverslips were coated with 2% polylysine solution before plating the cells in 12-well plates. HEK cells were transfected with 0.25 μg of pCAGGS-nsp1-Myc. Twenty-four hours post-transfection (unless otherwise specified), cells were washed twice with 500 μl 1× PBS and immobilized using 500 μl of 4% formaldehyde/in PBS for 10 min at room temperature. Cells were permeabilized with 500 μl of 0.5% Triton-X in 1× PBS to each well for 5 min at 4 °C. Cells were washed three times with 1× PBS and blocked with 200 μl of 1% bovine serum albumin (BSA) in PBS for 30 min at room temperature. Cells were incubated with 250 μl of 1:100 dilution of specified primary antibody in 1% BSA/1× PBS solution overnight at 4 °C. The antibody was discarded, and cells were washed with 1× PBS three times. Next, they were incubated with 250 μl of 1:400 dilution of the secondary antibody in 1% BSA/1× PBS solution for 40 min, followed by three 1× PBS washes. The coverslip was briefly dried before adding mounting media containing 4′,6-diamidino-2-phenylindole. Images were taken using a Leica microsystems TCS SP8 spectral confocal microscope using 40× magnification.

### HA-tag immunoprecipitation and HA peptide elution

In brief, HEK cells were grown under 5% CO_2_ at 37 °C to 70% confluency in 10 cm plates. Cells in each plate were transfected with 4.8 μg of respective DNA to express BioID2-fused nsp1 protein using Jet Optimus transfection reagent (Genesee Scientific). Twenty-four hours post-transfection, proteins were crosslinked with 0.1% formaldehyde for 10 min at 37 °C followed by quenching of extra formaldehyde with 12.5 mM glycine. For the pull down, about 2 × 10^7^ cells were washed with ice-cold PBS. The cells were collected and lysed with Thermo Fisher Pierce Cell lysis buffer (catalog no.: 87788), and the protein complex was immunoprecipitated using anti-HA magnetic beads (catalog no.: 88836) following the manufacturer's protocol. Beads were washed with 10 bead volumes of buffer five times using Pierce Tris-buffered saline buffer (catalog no.: 28360). The protein complex was eluted with 50 μl of 4 mg/ml Pierce HA peptide (catalog no.: 26184) at 37 °C for 15 min. Proteins were denatured using Laemmli buffer at 95 °C for 5 min before processing them for immunoblot.

### Isolation of SG core

Methods were adapted from the study by Jain *et al.* (50) to isolate SG core. In brief, HEK cells were grown under 5% CO_2_ at 37 °C to 70% confluency and in 10 cm plates. Cells in each plate were transfected with 4.8 μg of respective DNA to express nsp1-Myc protein using Jet Optimus transfection reagent (Genesee Scientific). Sixteen hours after transfection, cells were subjected to 30, 60, and 120 min of oxidative stress using 0.5 mM NaAsO_2_. Next, 4 × 10^7^ cells were collected, lysed in the 330 μl of lysis buffer (50 mM Tris–HCl, pH 7.4, 100 mM KOAc, 2 mM MgOAc, 0.5 mM DTT, 50 mg/ml heparin, 0.5% NP-40, 1 U/μl RNaseIN, and 1× protease inhibitor). The cytoplasmic fraction was isolated after centrifugation at 1000*g* for 5 min. Ten percent of the cytoplasmic fraction was collected (total), and the rest of the cytoplasmic fraction was further centrifuged at 18,000*g* to collect the SG core pellet. Ten percent of the supernatant was collected for running in the gel. The SG core was washed twice using the lysis buffer and resuspended in Laemmli buffer for subsequent immune blot analysis.

## Data availability

The MS proteomics data have been deposited to the ProteomeXchange Consortium *via* the PRIDE (59) partner repository with the dataset identifier PXD025980.

## Supporting information

This article contains supporting information (60, 61, 67).

## Conflict of interest

The authors declare that they have no conflicts of interest with the contents of this article.
