# Supplementary material for: Proximity-dependent biotinylation detects associations between SARS coronavirus nonstructural protein 1 and stress granule–associated proteins
Source: J Biol Chem. 2021 Nov 11;297(6):101399. doi: 10.1016/j.jbc.2021.101399 (PMC8580555; doi:10.1016/j.jbc.2021.101399)
Supplement: Table S6 — and Figures S1–S4 [file mmc2.docx]

**Proximity-dependent biotinylation detects associations between SARS coronavirus nonstructural protein 1 and stress granule-associated proteins**

Yevgeniy A. Gerassimovich^1^, Samantha J. Miladinovski-Bangall^1^, Kaitlin M. Bridges^1^, Linkel Boateng^2^, Lauren E. Ball^3^, Homayoun Valafar^2^ and Anita Nag^1^*

^1^Natural Sciences and Engineering, University of South Carolina Upstate, Spartanburg, SC 29303

^2^Department of Computer Science and Engineering, University of South Carolina, Columbia, SC 29208

^3^Department of Cell and Molecular Pharmacology, Medical University of South Carolina, Charleston, SC 29425

Materials included:

Supplemental Figure 1

Supplemental Figure 2

Supplemental Figure 3

Supplemental Figure 4

Supplemental Table 6


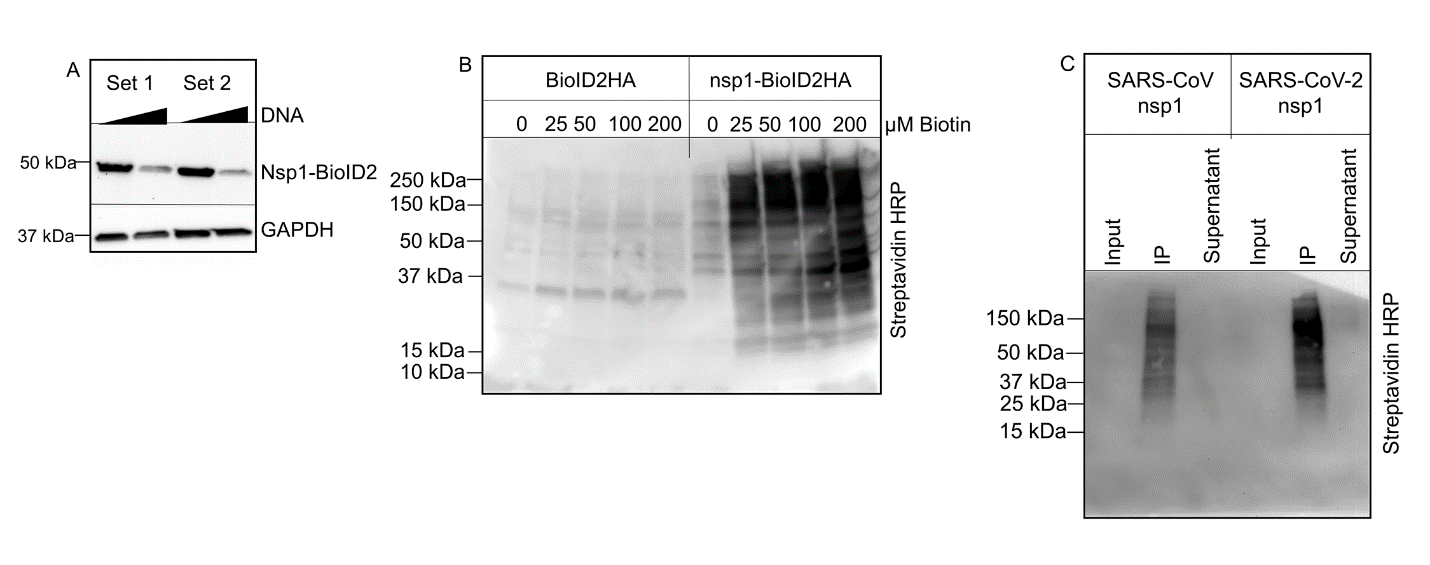


Supplemental Figure 1: Optimization of proximity-dependent biotinylation by nsp1. (A) Expression of SARS-CoV-2 nsp1 fused to BioID2. (B) Optimization of biotin concentration used for treating cells. Extracted proteins were run on an SDS-PAGE followed by western blot using streptavidin HRP. (C) Optimization of affinity purification (IP) showing all of the biotinylated protein was isolated.


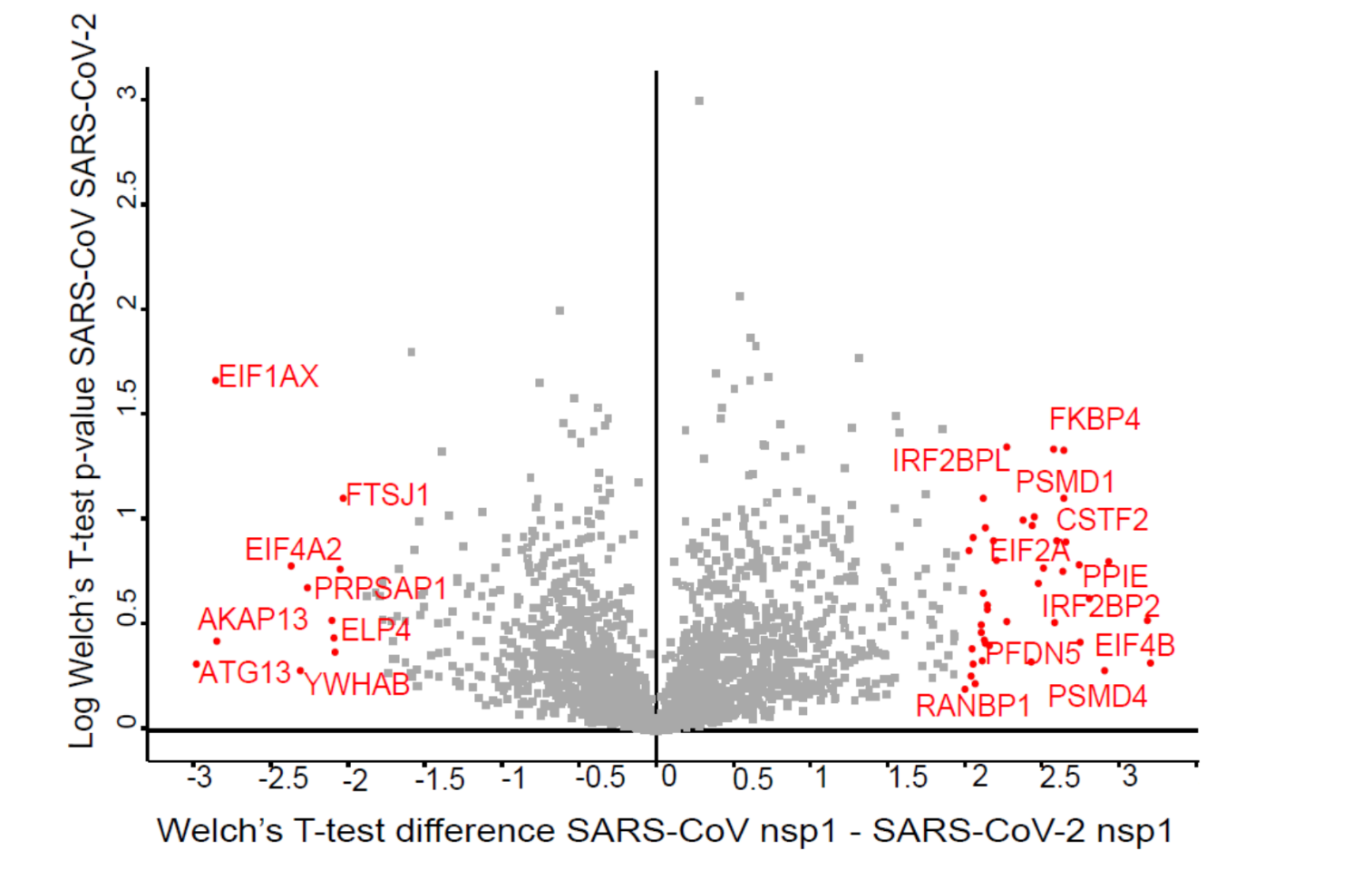


Supplemental Figure 2: Volcano plot comparing nsp1 of SARS-CoV and SARS-CoV-2.


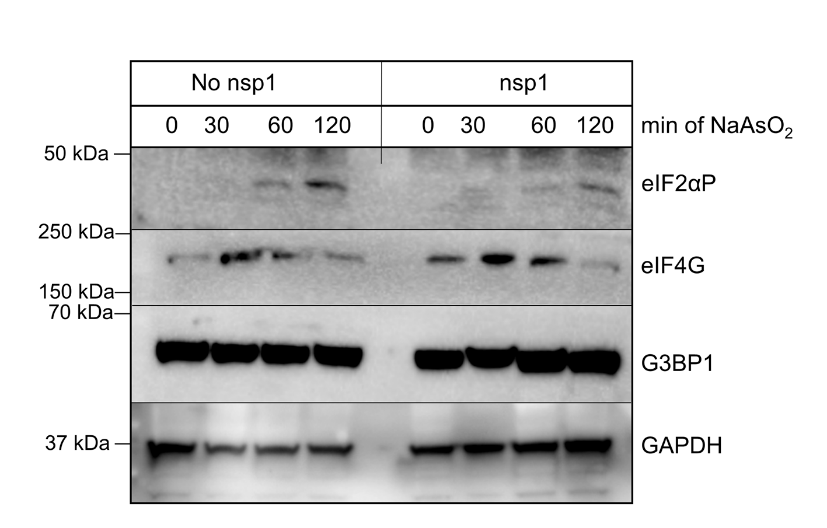


Supplemental Figure 3: Western blot of phosphorylated eIF2α, eIF4G, G3BP1 and GAPDH (control) after 0, 30, 60 and 120 minutes of NaAsO_2_ treatment.


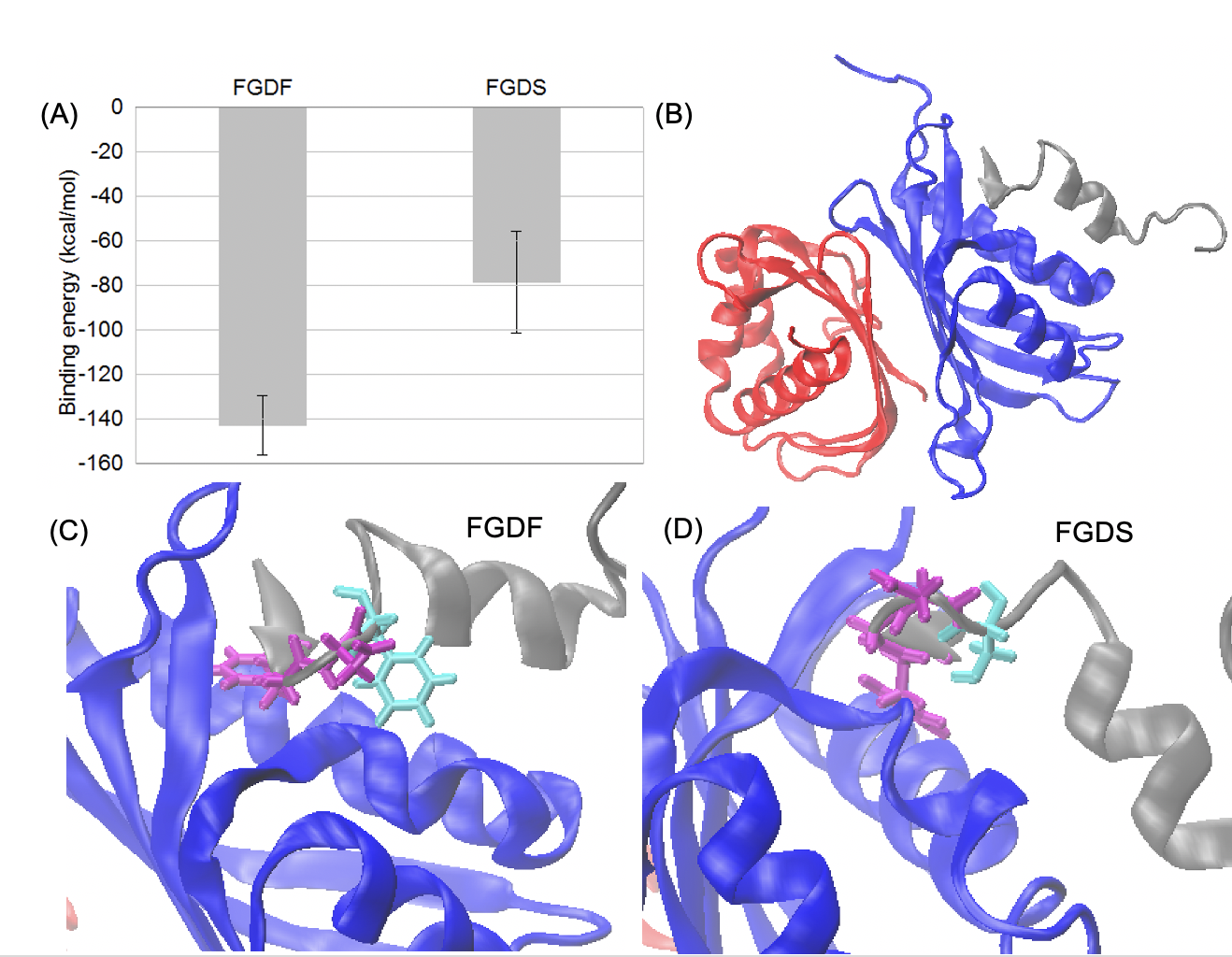


Supplemental Figure 4: Molecular dynamics simulation of binding of FGDF and FGDS peptides with G3BP1. (A) Energy calculation comparing binding of FGDF and FGDS sequences with G3BP1. Binding energy was computed using NAMDEenergy in VMD. (B) The simulation was conducted with the crystal structure of G3BP1 interacting with nsP3 of SFV (PDB ID: 5FW5). Binding of G3BP1 to (C) FGDF and (D) FGDS.

Supplemental Table 6

| AN365 | tgcgtggcttcgggCGctctgtggaagagg | nsp1 D33R mutation |
| --- | --- | --- |
| AN366 | cctcttccacagagCGcccgaagccacgca |  |
| AN367 | tgtggtctagtagagctggaaGaGggcgtactgcccc | nsp1 K58E mutation |
| AN368 | ggggcagtacgccCtCttccagctctactagaccaca |  |
| AN369 | agaaatggacggcattcagtacggtGAGagcggtataacactgg | nsp1 R99E mutation |
| AN370 | ccagtgttataccgctCTCaccgtactgaatgccgtccatttct |  |

**Methods: Molecular dynamics simulation**

The interactions of G3BP with wild-type and mutated SFV nsP3 FGDF were studied using molecular dynamics simulations using the crystal structure of human G3BP1 in complex with Semliki Forest Virus nsP3 (PDB ID: 5FW5). The mutated structure was created using the VMD [1] mutate function to replace Phe with Ser in the FGDF sequence. The mutated structure was minimized to remove bad contacts and optimize the structure. The CHARMM force field was used to describe atomic interactions and simulations were conducted using NAMD [2] MD package. The wildtype (FGDF) and mutated (FGDS) structures were both solvated with TIP3P water molecules. The solvated systems were initially minimized, followed by a series of 1 ns NVT and NPT equilibration simulations. During the equilibration process, the protein molecules were restrained to equilibrate the system to a temperature of 310 K and a pressure of 1 bar. The Langevin thermostat was used for temperature control and the Berendsen's barostat was used to couple the system pressure. The Particle Mesh Ewald [3] was used to compute the long-range electrostatic interactions with a 12 Å cutoff radii for short-range Van der Waals and Coulombic interactions. All bonds involving hydrogen atoms were fixed to their equilibrium value using SHAKE restraints. A time step of 2 fs was used in the simulations with periodic boundary conditions in all directions. A production simulation of 100 ns was performed to quantify the binding of FGDF and FGDS with G3BP in aqueous solution. Pair G3BP interactions with FGDF and FGDS were computed using the NAMDEnergy function in VMD.

1. Humphrey, W., A. Dalke, and K. Schulten, *VMD: visual molecular dynamics*. 1996, Journal of molecular graphics. p. 33-38.

2. Phillips, J.C., et al., *Scalable molecular dynamics with NAMD.* Journal of Computational Chemistry, 2005. **26**(16): p. 1781-1802.

3. Hockney, R.W. and J.W. Eastwood, *Computer simulation using particles*. 1988: Taylor & Francis Group. 564.
